# Letter from the Editor-in-Chief

**DOI:** 10.19102/icrm.2017.081005

**Published:** 2017-10-15

**Authors:** Moussa Mansour


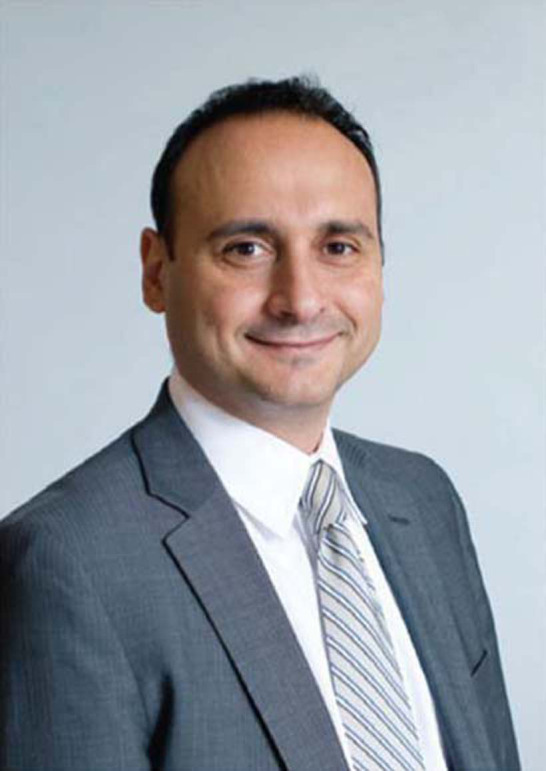


Dear Readers,

There have been significant advances in the treatment of patients with heart failure and left ventricular dysfunction. As a result, patients with low ejection fraction are living longer, and often experience the manifestations of low ejection fraction, including ventricular tachycardia and fibrillation. Not uncommonly, these patients present with electrical storm, which can be challenging to manage.

This issue of *The Journal of Innovations in Cardiac Rhythm Management* contains an important review article by Muser et al. titled “Electrical Storm in Patients with Implantable Cardioverter-Defibrillators: A Practical Overview.” The authors review the definition of electrical storm, discuss its incidence and predictors, and describe different available treatment strategies including pharmacologic therapy, catheter ablation, and sympathetic denervation. They also discuss the PAINESD score that accounts for the presence of several co-morbidities as a means to identify high-risk patients in whom prophylactic mechanical support may improve outcomes.

The significance of this article in the context of clinical practice is that it highlights the complexities surrounding the treatment of patients with electrical storm, especially during catheter ablation. Many studies have demonstrated the efficacy of catheter ablation in the setting of electrical storm and its role in reducing future recurrences. However, performing this procedure in acutely decompensated patients can be associated with significant complications. Collaboration between members of cardiac electrophysiology, interventional cardiology, heart failure, and intensive care is necessary. For us electrophysiologists, the care of these patients extends far beyond the electrophysiology laboratory, as many of them end up on mechanical support for several days after ablation. In some patients, sympathetic denervation may be needed, and being able to work successfully with skilled anesthesiologists and thoracic surgeons is important. Thus, the use of a multidisciplinary approach involving all of the above-mentioned specialties is critical to ensure the best possible outcome.

I hope that you enjoy reading this issue of *The Journal of Innovations in Cardiac Rhythm Management.*

Sincerely,


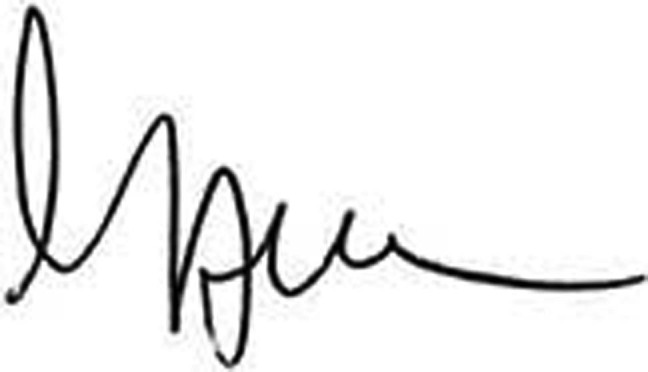


Moussa Mansour, MD, FHRS, FACC

Editor-in-Chief

The Journal of Innovations in Cardiac Rhythm Management

MMansour@InnovationsInCRM.com

Director, Atrial Fibrillation Program

Massachusetts General Hospital

Boston, MA 02114

